# Supraglottic jet oxygenation and ventilation saved a patient with ‘cannot intubate and cannot ventilate’ emergency difficult airway

**DOI:** 10.1007/s00540-016-2279-x

**Published:** 2016-11-15

**Authors:** Qiaoyun Li, Ping Xie, Benjun Zha, ZhiYun Wu, Huafeng Wei

**Affiliations:** 1 Department of Anesthesiology, The 180th Hospital of PLA, 180 Garden Road, FengZe District, Quanzhou, 362000 FuJian Province China; 2 Department of Anesthesiology and Critical Care, Hospital of the University of Pennsylvania, Philadelphia, 19104 Pennsylvania USA

**Keywords:** Jet ventilation, Supraglottic, Emergency, Difficult airway

## Abstract

The emergency difficult airway with the ‘cannot intubate and cannot ventilate’ (CICV) situation contributes to a high percentage of anesthesia- and emergency medicine-related morbidity and mortality. A new technique of supraglottic jet oxygenation and ventilation (SJOV) via the nasal approach was successfully used in an emergency to save a patient with a CICV difficult airway from a catastrophic outcome.

## Introduction

Emergency difficult airways with the ‘cannot intubate and cannot ventilate’ (CICV) situation may account for up to 25–28% of patient deaths in anesthetic practice [1, 2]; that statistic may be even worse in emergency and critical care medicine [2, 3]. The maintenance of adequate oxygenation and/or ventilation during difficult airway management is the foremost criterion in minimizing hypoxia-mediated morbidity and mortality [4]. Although many airway devices and methods have been developed to improve the safe practice of airway management, relatively few are designed to enhance oxygenation and ventilation during airway management, especially during direct laryngoscopy or fiberoptic intubation [4]. Supraglottic jet oxygenation and ventilation (SJOV) using WEI Nasal Jet Tube (Wei Nasal Jet or WNJ, Well Lead Medical Equipment Ltd., Guangzhou, China) and its equivalent assembly have been demonstrated to provide adequate oxygenation/ventilation in a morbidly obese patient with severe respiratory depression due to continuous intravenous propofol infusion [5]. In this paper, we report a case in which SJOV via the nasal approach maintained adequate oxygenation and ventilation for 30 min in CICV difficult airway management. This new technique saved the patient from an otherwise catastrophic outcome and avoided an invasive tracheostomy.

## Case report

A 34-year-old female weighing 41 kg was scheduled for a right thyroidectomy. The patient had slight scoliosis of the thoracic and lumbar spine and had bilateral weakness in her lower extremities. She had difficulty walking due to sequelae of poliomyelitis. Upon physical examination, a 5 × 5 cm lump on the right thyroid and a trachea deviation to the left was observed. A CT scan of the neck region demonstrated a compressed trachea. Airway examination showed limited neck extension, a two-finger-width mouth opening due to mandibular retraction, a small jaw, and a Mallampatti score of III. The patient showed no respiratory distress preoperatively.

A 0.3 mg intramuscular injection of scopolamine was given before anesthesia induction; the patient was well pre-oxygenated. Baseline vital signs were HR 70 beats/min, BP 110/68 mmHg, and SpO_2_ 98%. General anesthesia was induced with intravenous fentanyl 0.1 mg, propofol 80 mg, and cis-atracurium 7 mg. Mask ventilation could be easily performed. The initial intubation was performed with a video laryngoscope (Zhejiang UE Medical Corp, Zhejiang Province, China), which showed a very anterior position of the glottis, with only partial visibility of the epiglottis (III–IV Cormack view). Intubation was attempted twice with a 6.5-mm ID endotracheal tube but failed both times and resulted in moderate airway bleeding. Mask ventilation became difficult when the tidal volume was reduced to approximately 200 ml. A senior anesthesiologist then tried intubation with a light wand twice, but this method also failed. Mask ventilation then became impossible. A #3 laryngeal mask airway (LMA) was urgently placed but without successful ventilation. SpO_2_ dropped to 50% at this time and the surgeon was called to perform emergency tracheostomy.

**Fig. 1:**
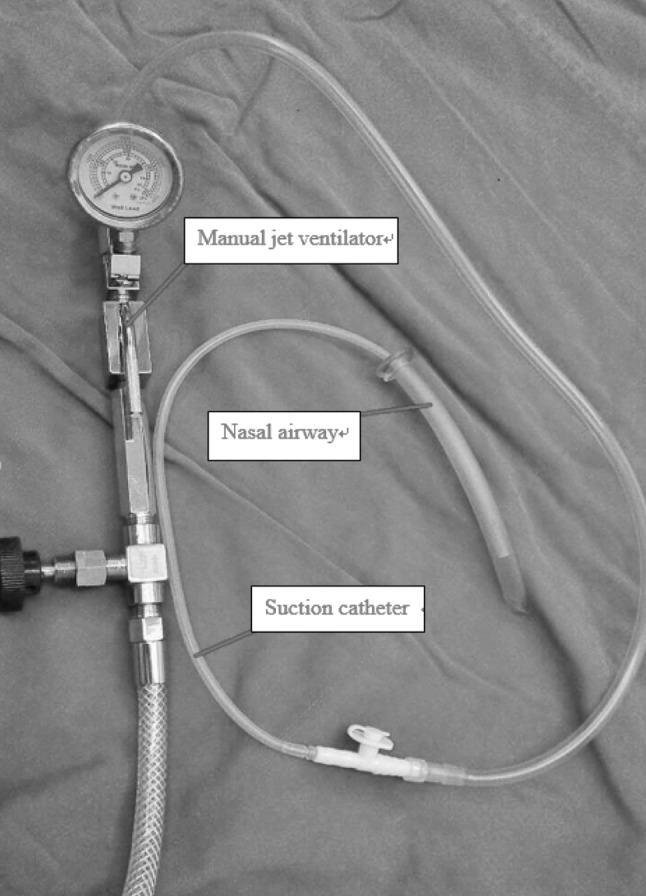
Jet nasal airway apparatus

Meanwhile, SJOV provided by the assembly shown in Fig. 1 was initiated as described previously [6] by using a manual jet ventilator (Well Lead Medical Equipment Ltd., Guangzhou, China; driving pressure 30 psi, breathing rate 60/min, I/E ratio 1:3) connecting a suction catheter (SuZhou City HyaHao Medical Instrument Ltd., SuZhou, China) through a regular nasal airway (Kendall, a division of Tyco Healthcare Group, NY, USA). The depth of the suction catheter used as a jet catheter was adjusted inside the oral pharyngeal area until maximum chest rise was achieved. SpO_2_ started to rise at 30 s after initiation of SJOV and improved from 10% (BP 89/63 mmHg, HR 124/min, ECG sinus tachycardia) to 85% within 3 min (BP 98/57, HR 82/min, ECG sinus rhythm). SpO_2_ reached 90% when the surgeons were ready to perform emergency tracheostomy. The decision was then made not to perform tracheostomy and to keep SJOV. SpO_2_ was kept above 90% and spontaneous breathing was recovered in 30 min under sedation with continuous infusion of propofol (4 mg/kg/h). The patient regained consciousness at 45 min from initiation of SJOV. Thereafter, SJOV was discontinued and the patient recovered in the post-anesthesia care unit without significant complications. The patient complained of sore throat and swallowing and eating difficulty for only 3 days postoperatively. On the fifth day in the hospital, an awake fiberoptic intubation went smoothly and was successfully performed under sedation with remifentanil and topical oral pharyngeal analgesia using a video laryngoscope. A right thyroidectomy was performed successfully without perioperative complications.

## Discussion

The case demonstrated striking results using SJOV to save a patient from a CICV emergency difficult airway, even with failed LMA. Although its mechanisms are not fully clear, the suction catheter used as a jet catheter with a small internal diameter (~2 mm) may have greater maneuverability to pass through the narrow airway secondary to the airway edema and place its distal end near the vocal cord opening. The high pulse pressure oxygen supplied by SJOV may inject some oxygen into the vocal cord opening more efficiency than just an oxygen insufflation or mask ventilation. Thus, the presence of a high concentration of oxygen around the vocal cord may better oxygenate patients with apnea by augmenting oxygen diffusion. However, it is possible that the oxygen level can be increased and maintained within the physiological range but with accumulation of CO_2_ as well. Nevertheless, it is critical to keep a relatively high oxygen level to prevent hypoxia-mediated brain death during emergency difficult airway management. Transient hypercapnia even with PaCO_2_ as high as 501 mmHg was reported without brain damage [6].

According to the ASA guidelines on difficult airway management, emergent transtracheal jet ventilation (TTJV) or tracheostomy should be the last step if the LMA placement fails to ventilate the patient in a CICV clinical scenario like that in the present paper [4]. However, compared to SJOV [5, 7–10], TTJV is more invasive and may be associated with higher incidence of barotrauma [11, 12]. Furthermore, it would have been difficult to perform TTJV in this patient due to the large thyroid goiter. Similarly, tracheostomy may require more preparation time due to waiting for the arrival of surgeons. The long waiting time would aggravate hypoxia and brain damage even with a successful tracheostomy [1, 2].

This patient had multiple implications for a difficult intubation based on her history and airway examination. Succinylcholine could not be used due to the chronic weakness of the lower extremities, while long-term muscle relaxant would not have been adequate due to the status of possible difficult airway. The safest approach would have been awake fiberoptic intubation, which was not used due to lack of experience. It has been reported that 14% of patients who suffered from severe complications from failed airway management were clearly indicated to receive awake fiberoptic intubation [1, 2]. It is prudent and safe to use awake fiberoptic intubation in those patients with clear implications for extreme difficult intubation, even if it may take a longer time and be uncomfortable. Another lesson we learned is that repetitive direct laryngoscopy could aggravate airway edema and bleeding [1, 3]. Alternative backup approaches should be considered to avoid using the same approaches repetitively whenever possible. It was lucky that the second author (Dr. Ping Xie) of the present study had learnt the technique of SJOV via nasal assembly (Fig. 1) from a national airway workshop in China just two months previously.

Compared to conventional infraglottic jet ventilation such as TTJV, SJOV via nasal assembly (like that in this case using the same principle of WNJ) has the following advantages: (1) it is relatively non-invasive; (2) it is quick to set up and easy to learn during elective cases (Fig. 1); (3) it can convey oxygen towards the vocal cord opening because the small jet catheter can be conveniently moved in and out towards the vocal cord opening as much as possible; and (4) the opening of the mouth and nose keep the SJOV in a well-maintained open system, thus minimizing the chance of barotrauma by avoiding injection of high-pressure gas into a sealed tissue pocket [11, 12].

However, SJOV in emergency difficult airway management may have the following disadvantages: (1) insufficient oxygenation/ventilation if the vocal cords are fully closed by laryngospasm or severe swelling; (2) PetCO_2_ may not be easily monitored; and (3) SJOV may not be suitable for long-term use considering the migration of the jet catheter away from the vocal cord opening, although this case used SJOV to maintain oxygenation/ventilation for about 1 h. Overall, SJOV is recommended to be used to augment the oxygen supply for all kinds of airway management, which should be a principle to follow in all difficult airway management guidelines [4, 13, 14]. SJOV may be much easier and safer to use than TTJV because it will not cause significant incidence of barotraumas [12].

LMA placement is the recommended approach before emergency surgical airways [4, 15, 16], but it may fail due to airway edema or bleeding, as in this patient. By comparison, a small ID jet catheter may pass through the tongue base or pharyngeal area and reach the vocal cord opening and supply high-pressure pulse oxygen flow into the lungs. It should be noted that although the SpO_2_ may be adequately maintained using SJOV, mild to moderate CO_2_ accumulation may happen. The closer the distal end of the jet catheter to the vocal cord opening, the better the chest rising and ventilation. Needless to say, PetCO_2_ or PaCO_2_ should be monitored whenever possible. In the case of airway bleeding, the opening feature of SJOV may facilitate the suction of blood and secretion and improve the oxygenation and ventilation simultaneously. In summary, pending the approval of the effectiveness of SJOV and acknowledgement of associated complications, this new technique seems to be an excellent method of maintaining adequate oxygenation and ventilation during difficult airway management by minimizing the morbidity and mortality associated with hypoxia and/or hypercapnia. It is our hope that WNJ, designed to provide SJOV more conveniently than this case’s current assembly, will contribute to the advanced use of SJOV in difficult airway management. Pending future clinical use and studies, SJOV may become a useful measure in future difficult airway management.
